# A comparative three-dimensional analysis of skeletal and dental changes induced by Herbst and PowerScope appliances in Class II malocclusion treatment: a retrospective cohort study

**DOI:** 10.1186/s40510-025-00571-5

**Published:** 2025-07-03

**Authors:** Eduardo Caleme, Alexandre Moro, Claudia Mattos, José Miguel, Klaus Batista, Jeanne Claret, Gaëlle Leroux, Lucia Cevidanes

**Affiliations:** 1 Universidade Positivo, Curitiba, Brazil; 2 Federal University of Paraná, Curitiba, Brazil; 3 University of Michigan–Ann Arbor, Ann Arbor, USA; 4 Fluminense Federal University, Niterói, Brazil; 5 Rio de Janeiro State University, Rio de Janeiro, Brazil

**Keywords:** Functional appliances, Herbst miniscope appliance, PowerScope appliance, Class II malocclusion treatment, Cone-Beam computed tomography (CBCT)

## Abstract

**Background:**

Skeletal Class II malocclusion is commonly treated using mandibular advancement appliances during growth. Evaluating the comparative effectiveness of different appliances can help optimize treatment outcomes.

**Objectives:**

This study aimed to compare dental and skeletal outcomes of Class II malocclusion treatment using Herbst and PowerScope appliances in conjunction with fixed orthodontic therapy.

**Methods:**

This retrospective comparative study included 46 consecutively treated patients in two university clinics: 26 with PowerScope and 20 with Herbst MiniScope. CBCT scans were obtained before and after treatment. Skeletal and dental changes were analyzed using maxillary and mandibular voxel-based regional superimpositions and cranial base registrations, aided by AI-based landmark detection. Measurement bias was minimized through the use of a calibrated, blinded examiner. No patients were excluded from the analysis. Due to the study’s retrospective nature, no prospective registration was performed; the institutional review board granted ethical approval.

**Results:**

The Herbst group showed greater anterior displacement at B-point and Pogonion than PowerScope (2.4 mm and 2.6 mm, respectively). Both groups exhibited improved maxillomandibular relationships, with PowerScope’s SNA angle reduced and Herbst’s SNB increased. Vertical skeletal changes were observed at points A, B, and Pog in both groups. Herbst also resulted in less lower incisor proclination and more pronounced distal movement of upper incisors.

**Conclusion:**

Both appliances effectively corrected Class II malocclusion. Herbst promoted more pronounced skeletal advancement, while PowerScope induced greater dental compensation. These findings may be generalizable to similarly aged Class II patients in CVM stages 3–4.

## Background

Class II malocclusion represents a multifaceted orthodontic challenge attributable to various dentoskeletal anomalies. Such malocclusions may arise from malpositioning of the maxillary or mandibular dentition, posterior displacement of the mandible, small mandible relative to the maxilla, anterior displacement of the maxilla, or a combination thereof. Notwithstanding the multifactorial etiology of Class II malocclusion, mandibular retrognathism is frequently identified as a predominant contributing factor, thus representing an essential obstacle to effective management [1, 2].

Among functional appliances, the Herbst appliance has emerged as a particularly prevalent choice for the correction of Class II discrepancies [3]. Despite potential patient discomfort, this constraint facilitates a continuous anterior positioning of the mandible, promoting anterior displacement of the condyle within the articular fossa and enhanced condylar growth. Importantly, the efficacy of the Herbst appliance is not contingent upon patient compliance. This feature is particularly advantageous during the pubertal growth spurt when the effects of the appliance are ostensibly maximized [3]. The Herbst appliance has gone through numerous changes over the years to enhance patient comfort, ensure appliance stability, and minimize breakage [4]. One of the newest versions is the Herbst MiniScope, equipped with a telescopic system that does not disengage. The upper piston is positioned mesially relative to the molar, facilitating easier placement and adjustments. Furthermore, the Apple-Core screw, used in conjunction with the MiniScope system, permits complete mandibular movement in the sagittal and frontal planes [4]. This increases patient comfort, reduces complications, and improves device acceptance.

Notably, there is a paucity of studies addressing the configuration of the Herbst appliance under consideration, thereby emphasizing the significance of the present study.

PowerScope is a contemporary derivative of the Herbst appliance, engineered to address clinicians’ demands for a more patient-friendly, easily installable, and maintenance-efficient appliance [3, 5, 6]. Unlike the Herbst appliance, PowerScope is flexible, allowing for greater mandibular mobility, thus enhancing patient comfort. PowerScope features a mechanism for direct attachment to the orthodontic archwires, obviating the need for stainless steel crown anchorage and streamlining the installation process. PowerScope employs a telescopic assembly with a NiTi spring, exerting a constant force of 260 g [3]. Notably, PowerScope does not lead to the removal of the condyle from the articular fossa, thus preventing actual mandibular propulsion.

While a limited body of research has employed 3D imaging modalities to assess the Herbst appliance [7–10] investigations [6, 11] addressing the PowerScope appliance have predominantly utilized 2D imaging techniques with inherent structural superimposition and magnification errors. The advent of voxel-based registration techniques for cone-beam computed tomography (CBCT) has further refined the accuracy of craniofacial superimpositions with the use of masks in anatomically stable regions to compare voxel intensity, thereby facilitating a superior alignment of images compared to observer-dependent methodologies [12]. These advancements in 3D imaging technologies enable a more comprehensive analysis of orthodontic treatment impacts on mandibular positioning and growth within the craniofacial complex.

## Objectives

By offering a focused analysis of the structural changes induced by the Herbst MiniScope and PowerScope appliances, this study seeks to contribute valuable insights into the comparative effectiveness of these treatment modalities, thereby guiding clinicians in the evidence-based selection of functional appliances for managing Class II malocclusion.

The primary aim of this study is to comprehensively examine the dental and skeletal three-dimensional outcomes of Class II malocclusion treatment using the Herbst MiniScope design and PowerScope appliances. The specific objectives of this study are: (1) to quantitatively analyze the skeletal changes induced by each appliance, focusing on mandibular advancement and the alteration of craniofacial structures; (2) to assess the dental changes that occurred with treatment, including changes in tooth angulation and linear displacements; and (3) to compare the efficacy of these appliances in achieving desired orthodontic and orthopedic corrections. The null hypothesis of this study is that both appliances produce similar levels of mandibular advancement. The reporting of the present study follows the Strengthening the Reporting of Observational Studies in Epidemiology statement.

## Materials and methods

### Study design

This multicenter, retrospective clinical trial was approved by the Institutional Review Board (HUM00251248) at the University of Michigan.

### Setting and participants

The study sample was retrospectively collected from the records of consecutively treated patients with skeletal Class II malocclusion across two university clinics. All patients received comprehensive orthodontic treatment using either the PowerScope (26 patients) or Herbst MiniScope (20 patients) fixed functional appliances, followed by treatment with fixed appliances. All treatments were performed by graduate students supervised by experienced orthodontists using standardized clinical protocols for each appliance. To qualify for the study, the following eligibility criteria had to be followed:

1. Available CBCT images before and after treatment.
2. Cervical vertebra maturation stage 3 or 4 [13].
3. Bilateral Class II molar relationship of at least half a cusp.
4. Convex facial profile, improved facial profile when the mandible was positioned forward.
5. Overjet ≥ 4 mm.
6. Absence of dental issues.

The number of patients with full cusp Class II and end-on Class II in each group was comparable, and so were the overjet, the amount of crowding in the individual arches, and the curve of Spee, all of which could affect incisor inclination and, consequently, the amount of mandibular advancement.

The Herbst group (*n* = 20) had a mean age of 11.2 years at treatment onset and 14.9 years at completion (Table 1). The PowerScope group (*n* = 26) averaged 12.1 years at onset and 15.5 years at completion. Class II correction duration was longer for the Herbst group (12 months) compared to PowerScope (5.8 months). At the start of treatment, all patients presented cervical vertebral maturation (CVM) stages 3 or 4, according to the protocol proposed by McNamara and Franchi [13]. 

**Table 1:** Sample description

Treatment	Herbst	PowerScope
	(*n* = 20)	(*n* = 26)
Age T1 (y)	11.2 ± 1.8	12.1 ± 1.4
Age T2 (y)	14.9 ± 2.8	15.5 ± 1.4
Gender (M/F)	10 M/10 F	9 M/17 F
CVM Stage	13 CVM 3/7 CVM 4	15 CVM 3/11 CVM 4
Functional Appliance Use (mo)	12.0 ± 1.3	5.8 ± 2.1

Values are presented as mean ± standard deviation

Patients underwent anamnesis, clinical evaluation, and CBCT scans before (T1) and after (T2) treatment; CBCT scans were acquired following a standardized imaging protocol: head positioned with the Frankfurt plane parallel to the ground, scanning time of 40 s, 170 × 170 mm field of view, and patients biting in centric occlusion. Following the ALADAIP principles [14], the i-CAT scanner (model 9140) with a voxel size of 0.3 mm was used. CBCT images were then exported as DICOM files.

**Fig. 1:**
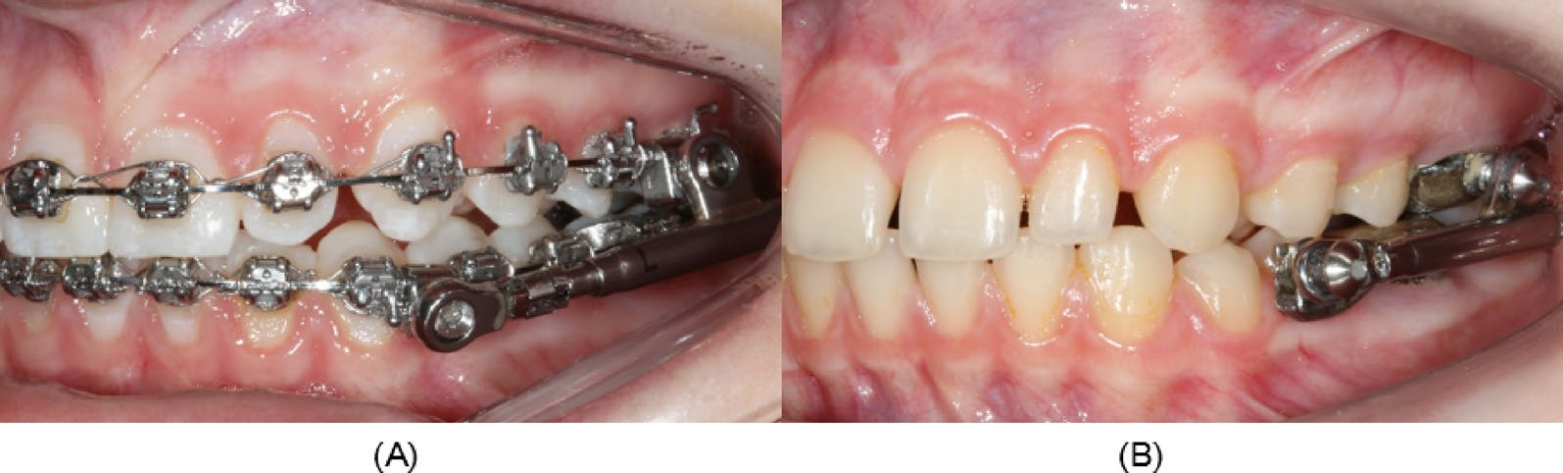
Appliances for correction of Class II malocclusion. **A** PowerScope. **B** Herbst

The treatment procedure for both groups consisted of fixed orthodontic treatment, with a 0.022-inch prescription with additional anchorage: a transpalatal arch for the maxillary molars and a lingual arch attached to the mandibular first molars. In the PowerScope (American Orthodontics, Sheboygan, WI) group, leveling progressed to a 0.019 × 0.025-inch stainless steel wire before appliance insertion, following the protocol proposed by Moro et al. [3] (Fig. 1A). The Herbst group underwent Class II correction with the MiniScope (American Orthodontics, Sheboygan, WI) design before comprehensive orthodontic treatment (Fig. 1B). Rollo bands (American Orthodontics, Sheboygan, WI) were cemented onto the four first molars, and a cantilever was placed on the mandibular molars. A construction bite registration was obtained for the edge-to-edge incisor relationship, with a mean mandibular advancement of 6.9 mm (max: 10 mm, min: 4 mm) in a single step. Patients in both groups wore Class II elastics during sleep for approximately 6 months after the use of Class II correctors to avoid relapse.

### Variables and measurement

**Fig. 2:**
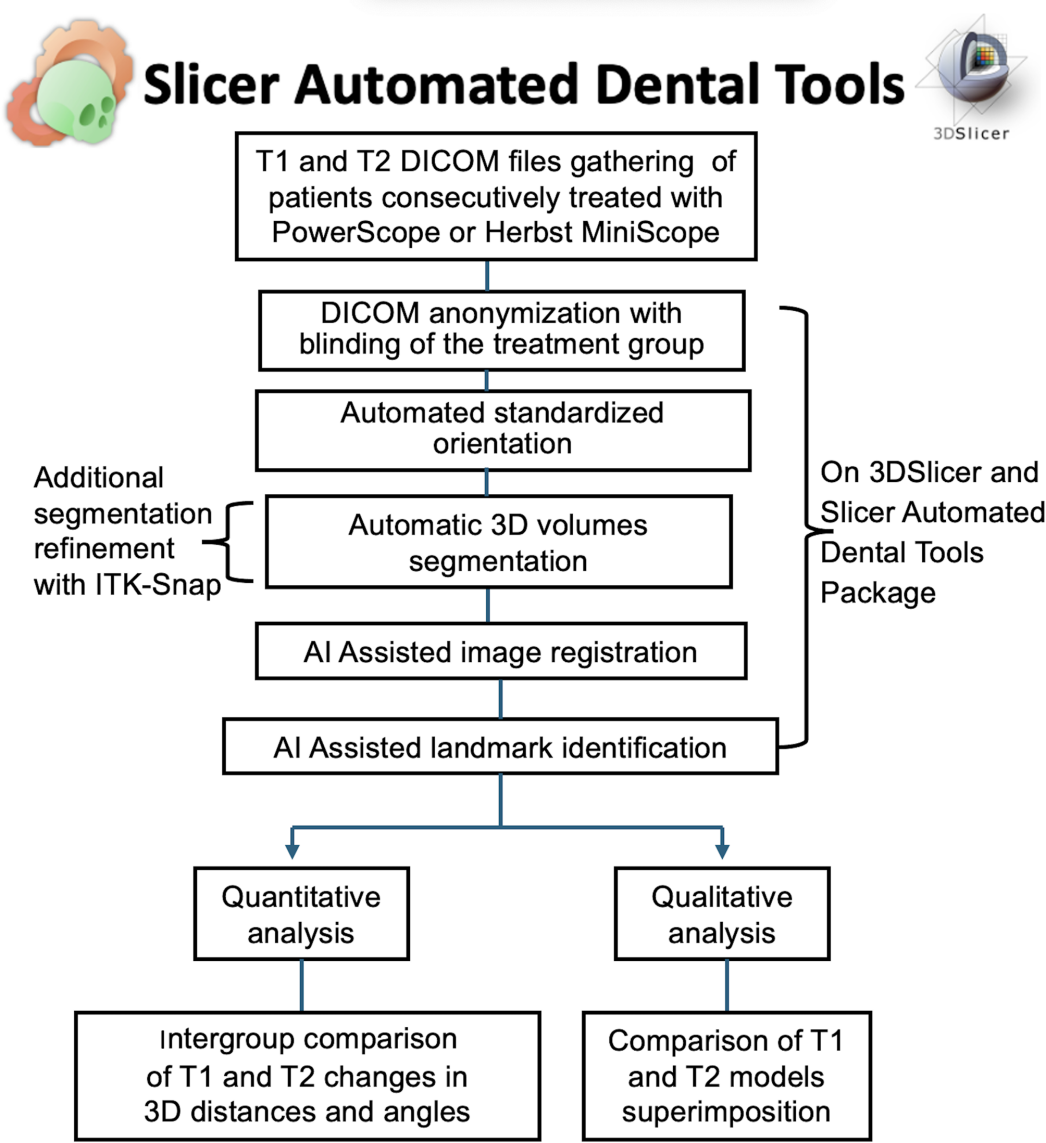
Workflow of the three-dimensional analysis. T1 - before treatment, T2 - at the end of comprehensive orthodontic treatment

Image analysis was performed by a single calibrated clinician using dedicated automated tools based on recently validated deep-learning approaches [15–17]. The DICOM files were anonymized, and the group information was then blinded by randomly assigning a number to each patient. All images were processed using the open-source 3DSlicer software (version 5.6.1, http://www.slicer.org), as illustrated in Fig. 2. Automated orientation of T1 was performed according to the Frankfurt and the midsagittal planes. The CBCT images taken after the comprehensive treatment were automatically aligned with the oriented T1 scans using voxel-based superimposition [15]. Stable reference areas for superimposition were used to assess overall facial changes, whereas regional mandibular and maxillary superimposition was employed to assess bone remodeling and tooth displacement. For the cranial base and mandibular superimpositions [15] the CBCTs were oriented according to the Frankfurt horizontal and midsagittal planes. In contrast, the maxillary regional superimposition [18] was oriented according to the occlusal and midsagittal planes.

Virtual Toolkit (vtk) models were created automatically using an artificial intelligence-based tool. Automated segmentation of the cranial base, maxilla, and mandible was achieved [16]. When required, the segmentation underwent additional refinement using the ITK-SNAP software (version 3.8.0; http://www.itksnap.org). Automated identification of landmarks was conducted to accurately predict the localization of the cephalometric landmarks essential for the analysis. All the landmarks were then manually controlled and adjusted (Table 2).

**Table 2:** Description of landmarks

Landmarks	Description
Cranial Base	
Sella (S)	The geometrical center of the Sella turcica
Nasion (N)	The most anteroposterior point at the frontonasal suture
Porion (Po)	The most anterosuperior point of the external acoustic meatus
***Maxillary skeletal***	
A point (A)	The deepest concavity between the ANS and U1R
Anterior nasal spine (ANS)	The most anterior point of the nasal spine
Posterior nasal spine (PNS)	The most posterior point of the nasal spine
***Maxillary dental***	
Maxillary incisor incisal edge (U1O)	The most central and occlusal point of the upper right central incisor tip
Maxillary incisor root apex (U1R)	The apex of the of the upper right central incisor root
Maxillary molar cusp tip (UR6O, UL6O)	The center of the mesiobuccal cusp of the upper first right and left molars
Maxillary molar root apex (UR6R, UL6R)	The apex of the mesiobuccal root of the upper first right and left molars
**Mandibular Skeletal**	
Menton (Me)	The most inferior point of the mandibular symphysis
Gnathion (Gn)	The most anteroinferior point of the mandibular symphysis
Pogonion (Pog)	The most anterior point of the mandibular symphysis
B-point (B)	The deepest concavity near the transversal midline at the anterior mandible
Gonion (Go)	Two points were placed (right and left Gonion) on the most lateral posterior inferior point at an angle of the mandible, constructed point perpendicular to the bisection of the ramus of the mandible and mandibular plane. Midpoint taken between 2 points
Condylion (Co)	Two points were placed (right and left Condylion) on the most lateral posterior superior point of the head of the condyle. Midpoint taken between 2 points
***Mandibular Dental***	
Mandibular incisor incisal edge (L1O)	The most central and occlusal point of the lower right central incisor tip
Mandibular incisor root apex (L1R)	The apex of the lower right central incisor root
Mandibular molar cusp tip (LR6O, LL6O)	The central point at the mesiobuccal cusp of the lower first right and left molars
Mandibular molar root apex (LR6R, LR6R)	The apex of the mesiobuccal root of the first right and left molars

For the quantitative analysis, the “Automatic Quantification of 3D Components (AQ3DC)” tool was used to calculate linear measurements in millimeters along the three coordinates: anteroposterior (x-axis), upper-lower (y-axis), and right-left (z-axis). Root mean square was used to calculate the 3D displacement. In addition, angular measurements in degrees were obtained for the three components: yaw, pitch, and roll. The analysis focused on comparing overall displacements from T1 to T2, both relative to the cranial base, as well as bone remodeling and dental changes relative to the maxilla and mandibular regional superimpositions.

For the qualitative analysis, the shape was analyzed by subtracting the point-based models of T1 and T2, allowing for better visualization of changes that occurred on the three spatial axes (x, y, and z). Treatment outcomes were highlighted using semitransparent overlays and colormaps generated automatically by the software. By adjusting the surface distance values on the color bar, the interpretation of the distance maps was enhanced, thereby improving the understanding of the magnitude of positional changes between the models. Dental extrusion, flaring, and bone growth were correlated with positive numbers, whereas bone resorption was associated with negative numbers.

The primary outcomes measured were the displacement of the Pogonion and B points. Secondary outcomes were the displacement of the A point, growth of the ramus and corpus of the mandible, mandibular rotation, and displacement of the first molars and incisors.

For the method error analysis, the same examiner performed the three-dimensional analysis of 10 patients (20% of the sample) twice, with a one-month interval between assessments.

### Bias

All image analyses were performed by a calibrated examiner blinded to group allocation at the measurement time. The DICOM files were anonymized, and patients were assigned random numbers to avoid recognition during assessment. Automated segmentation and landmark identification were performed using validated AI-assisted tools, reducing subjective input and enhancing measurement consistency. Manual refinement of landmarks was limited to minor corrections and was carried out according to a standardized protocol.

Selection bias was mitigated by the consecutive inclusion of patients meeting strict eligibility criteria. Both treatment groups were matched for cervical vertebral maturation (CVM stages 3–4), age, and the presence of puberty markers to control for the developmental stage at treatment onset.

### Study size

A sample size calculation was performed for the primary outcome of Pogonion changes, considering alfa = 0.05, power of 80%, a mean difference to be detected of 2 mm, and the standard deviation of a previous study [7]. The calculation indicated a need for at least 18 subjects in each group; 20 patients were included to account for attrition.

### Statistical analysis

Descriptive statistics (means and standard deviations) were calculated for the cephalometric variables on the T1 and T2 CBCTs in both groups. The normality of the data was assessed utilizing the Shapiro-Wilk test. Because the sample data did not have a normal distribution, descriptive statistics were presented through medians, interquartile ranges, and confidence intervals. Between-group differences were assessed by the Mann-Whitney test. The mean values of the between-group differences were calculated by subtracting the absolute mean values of each group. P-values below 0.05 were considered statistically significant. Statistical analysis was conducted using the Jamovi software [19] (version 2.3, Sydney, Australia).

## Results

### Participants and descriptive data

The final sample comprised 26 patients in the PowerScope group and 20 in the Herbst Miniscope group. Groups were similar regarding age, CVM stage, and treatment duration. However, gender distribution revealed a larger proportion of females in the PowerScope group than in the Herbst Miniscope group (Table 1). The intraexaminer reliability was evaluated with the intraclass correlation coefficient, demonstrating a good reliability of 0.998. No subgroup or adjusted analyses were performed.

### Outcome data, main results, and other analyses

Baseline measurements (Table 3) revealed no difference between groups that could cause biases in the analysis.

Changes are expressed in Horizontal (AP), vertical (SI), and 3D measurements. 3D distances are calculated according to the formula: B_T1_(x_1_,y_1_,z_1_) and B_T2_ (x_2,_ y_2_, z_2_) is given by: 3D distance B_T1_B_t2_ = √[(x_2_– x_1_)^2^ + (y_2_– y_1_)2 + (z_2_– z_1_) [2].

**Table 3:** Baseline descriptive statistics and comparison between the Herbst and the PowerScope groups

Variables	Herbst			PowerScope			Intergroup differences		
	Mean	Median	IQR	Mean	Median	IQR	Mean	95% CI	*p*-value*
SNA (°)	83.3	81.7	5.4	82.7	82.4	5.4	0.6	−3.0;2.0	0.868
SNB (°)	78.2	77.5	4.7	77.2	78.2	5.0	1.0	−3.2;1.8	0.489
SN-GoGn (°)	29.6	27.6	4.1	30.4	29.8	8.7	−0.8	−2.1;5.6	0.517
ANB (°)	5.2	5.0	1.9	5.5	5.3	2.7	−0.3	−0.9;1.8	0.561
PNS-ANS (mm)	49.9	49.3	6.9	49.9	49.6	3.9	0.0	−2.5;2.3	0.701
Co-Go-Gn (°)	117.0	116.0	7.7	115.0	116.0	6.0	2.0	−5.7;1.5	0.276
Co-Pog (mm)	94.7	94.7	10.4	94.4	94.9	5.6	0.3	−4.2;3.4	0.816
Co-Go (mm)	47.1	46.9	5.1	47.9	48.5	6.2	−0.8	−2.0;3.4	0.579
IMPA (°)	83.3	82.9	8.7	85.2	85.9	8.5	−1.9	−1.6;6.1	0.306

*Mann Whitney U test; statistical significance at *p* < 0.05

**Fig. 3:**
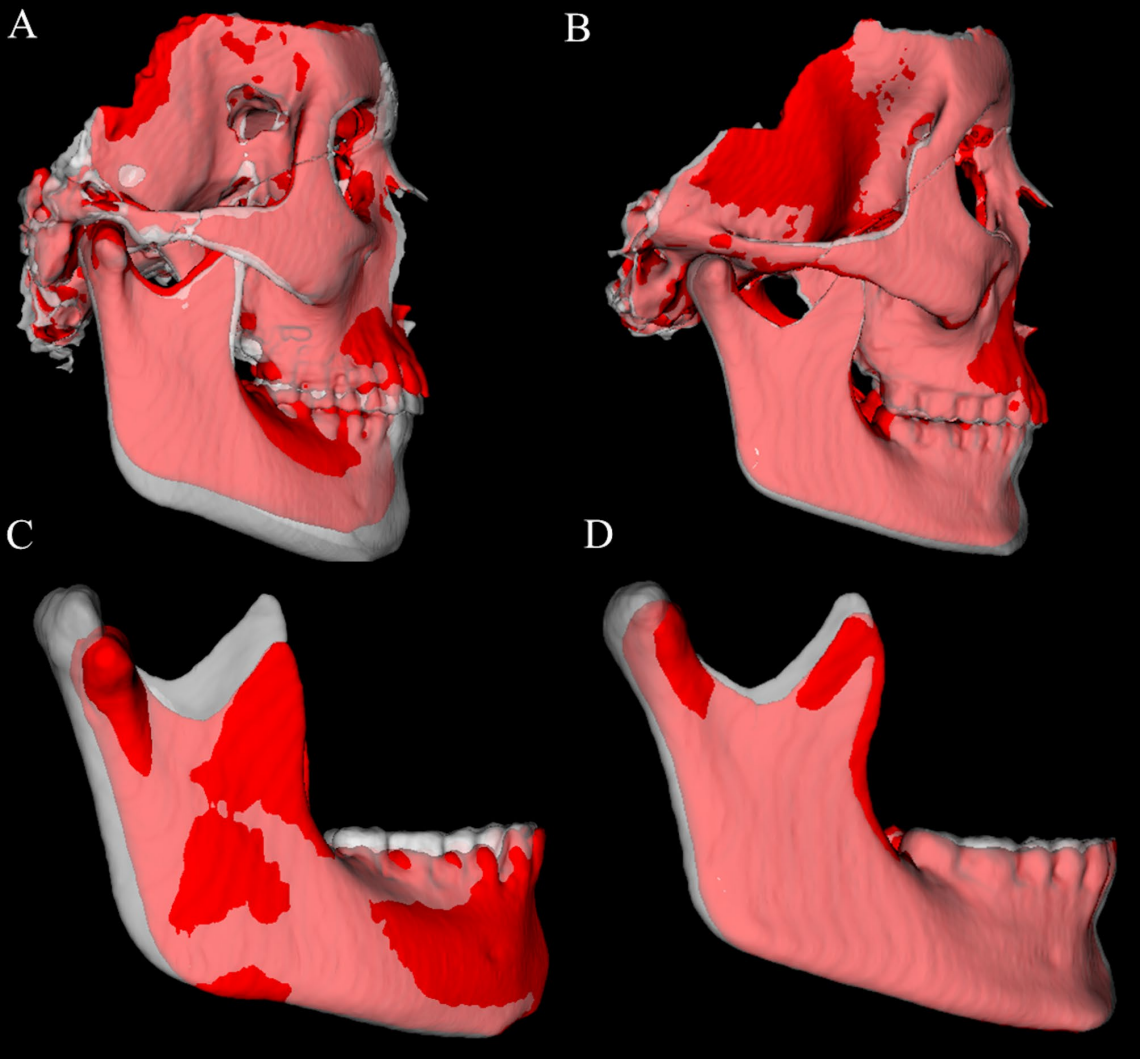
T1 (red) and T2 (semitransparent white) superimpositions for two patients with variable responses in the Herbst group. **A** and **B** cranial base superimposition of the T1 and T2 surface models of two patients treated with the Herbst MiniScope appliance. Note a more marked downward and forward displacement of the mandible relative to the cranial base in the patient in **A**; **C** and **D**, mandibular superimposition of the T1 and T2 surface models of two patients treated with Herbst MiniScope. Note a more marked ramus and condylar remodeling with postero-superior growth of the mandibular condyle in the patient in **C**. Note the differences of direction and amount of growth in two patients treated with Herbst MiniScope, as well as the individual variation in response to treatment

**Fig. 4:**
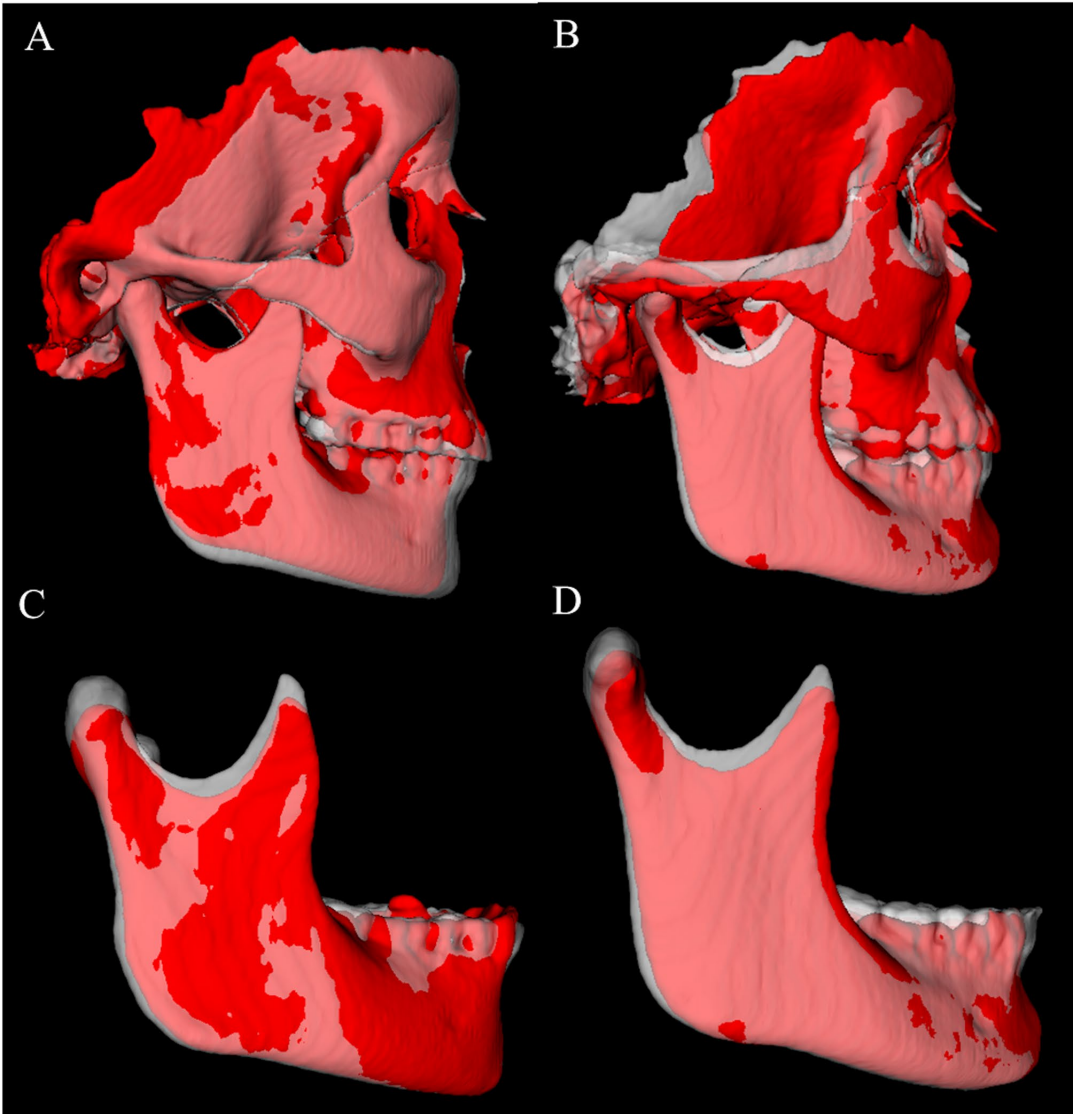
T1 (red) and T2 (semitransparent white) superimpositions for two patients with variable responses in the PowerScope group. **A** and **B**, cranial base superimposition of the T1 and T2 surface models of two patients treated with the PowerScope appliance. Note a more marked downward and forward displacement of the mandible relative to the cranial base in the patient in **A**; **C** and **D**, mandibular superimposition of the T1 and T2 surface models of two patients treated with the PowerScope appliance. Note a more marked ramus and condylar remodeling with postero-superior growth of the mandibular condyle in the patient in **C**. Note the differences of direction and amount of growth in two patients treated with the PowerScope appliance, as well as the individual variation in response to treatment

**Fig. 5:**
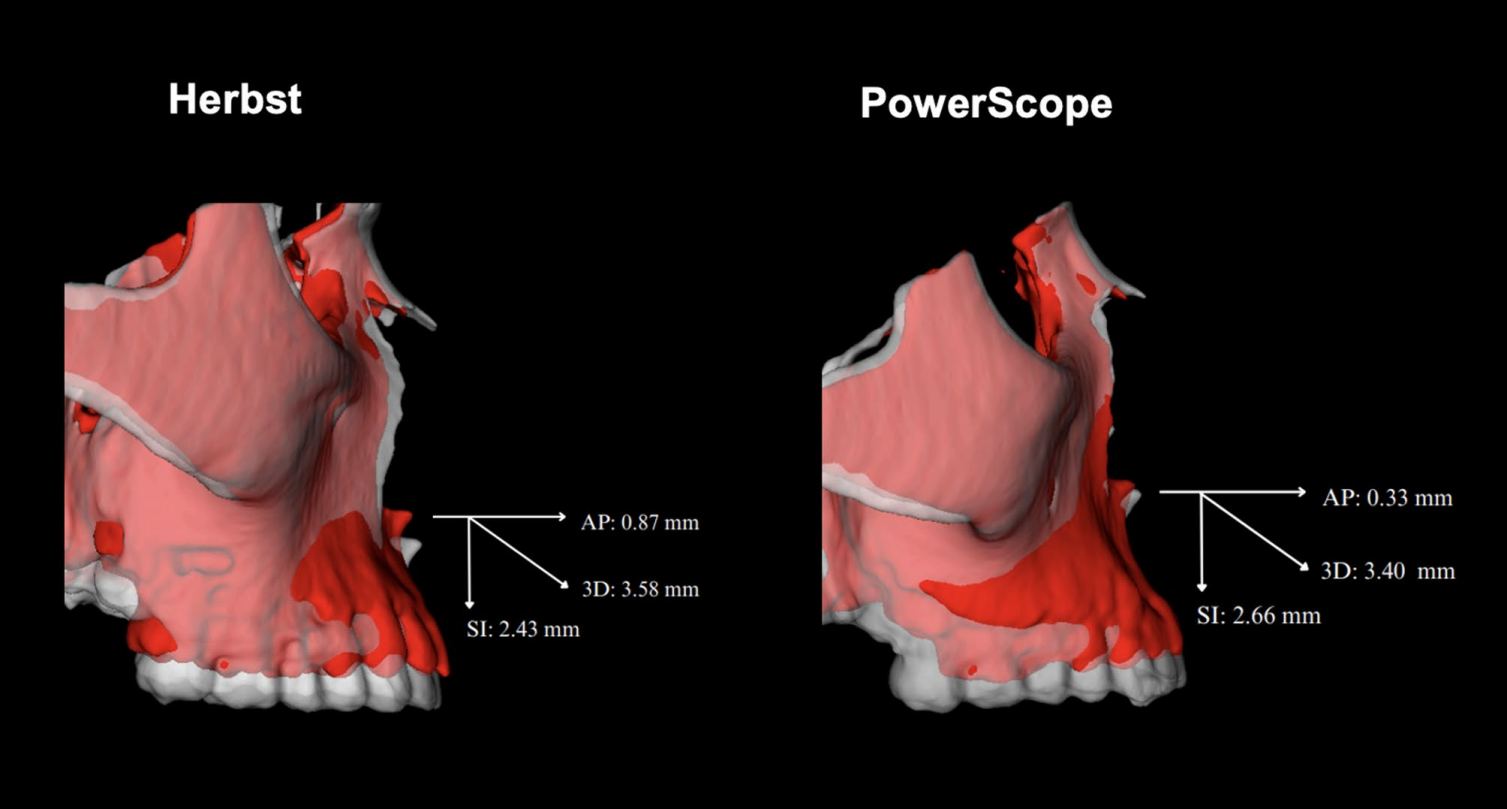
Comparison of facial changes (T1 to T2) relative to the cranial base for the Herbst and PowerScope groups in cases representative of the sample mean values for A-point displacement: AP = Anteroposterior; SI = Superoinferior

**Fig. 6:**
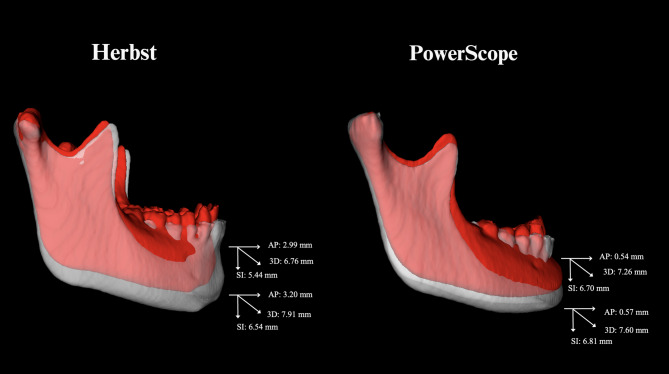
Comparison of facial changes (T1 to T2) relative to the cranial base for the Herbst and PowerScope groups in cases representative of the sample mean values for B and Pog points displacement: AP = Anteroposterior; SI = Superoinferior

Table 4; Figs. 3, 4, 5 and 6 present the overall facial changes between T1 and T2, based on scans registered concerning the cranial base superimposition. The anterior displacements of B-point and Pog were more significant in the Herbst group, respectively, on average 2.4 mm and 2.6 mm more anterior than in the PowerScope group. The maxillomandibular discrepancy (ANB) was decreased in both appliances, with no statistical difference. Both groups presented vertical displacement of points A, B, and Pog with no differences between the groups.

**Table 4:** Comparison of facial changes (T1 to T2) relative to the cranial base for the Herbst and PowerScope groups

Variables		Herbst			PowerScope			Intergroup difference		
		Mean	Median	IQR	Mean	Median	IQR	Mean	95% CI	*p*-value^*^
***Maxillary skeletal***										
A-point displacement (mm)	AP	0.87	1.05	1.97	0.33	0.15	1.45	0.54	−1.40;0;20	0.123
	SI	2.43	2.1	3.43	2.66	2.5	4.13	−0.23	−1.50;1.80	0.859
	3D	3.58	3.25	3.9	3.4	3.1	3.63	0.18	−1.80;1.20	0.579
SNA (°)		−0.49	−0.35	2.08	−0.77	−0.6	1.65	−0.28	−1.50;0.80	0.542
ANS-PNS (°)		0.6	0.2	2.15	1.38	1.35	2.1	−0.78	−0.09;1.50	0.082
***Mandibular skeletal***										
B-point displacement (mm)	AP	2.99	3.2	2.67	0.54	0.35	2.17	2.45	−3.89;−1.30	**< 0.001**
	SI	5.44	5.45	4.2	6.7	6.7	5.3	−1.26	−0.70;3.30	0.298
	3D	6.76	6.95	3.93	7.26	7.6	4.92	−0.5	−1.50;2.30	0.626
Pog displacement (mm)	AP	3.2	2.95	2.5	0.57	0.2	2.53	2.63	−4.00;−1.60	**< 0.001**
	SI	6.54	6.25	5.65	6.81	6.55	5.57	−0.27	−2.30;2.80	0.912
	3D	7.91	7	4.23	7.6	7	5.97	0.31	−2.90;1.80	0.748
SNB (°)		1.26	0.9	2	0.22	0.25	1.65	1.04	−2.00;0.10	0.069
SN-GoGn (°)		0.4	0.1	3.55	0.97	0.5	3.2	−0.57	−0.80;2.70	0.406
Go-Gn (°)		0.06	0.4	2.58	1.5	1.15	2.48	−1.44	−0.30;2.70	0.094
***Maxillomandibular***										
ANB (°)		−1.36	−1.3	1.87	−0.99	−0.9	1.38	0.67	−0.09;1.50	0.082

For linear measurements, a positive value indicates anterior displacement in the AP direction, whereas a negative value indicates superior displacement. In the SI direction

For angular measurements, a positive value indicates clockwise rotation, whereas a negative value indicates counterclockwise rotation

AP = Anteroposterior; SI = Supero inferior

*Mann Whitney U test was utilized to the assess difference between the two groups; statistical significance at *p* < 0.05

Table 5; Figs. 3, 4 and 8 present the mandibular skeletal and dental changes between T1 and T2, as measured in scans registered concerning the mandibular regional superimposition. There were no significant differences in mandibular skeletal changes between the groups. Regarding dental changes, the lower incisors presented more significant proclination in the PowerScope group, on average, 1.5 mm. The lower first molars tipped significantly on both sides.

**Table 5:** Comparison of mandibular skeletal and dental changes (T1 to T2) for the Herbst and the PowerScope groups

Variables		Herbst			PowerScope			Intergroup difference		
		Mean	Median	IQR	Mean	Median	IQR	Mean	95% CI	*p*-value^*^
***Mandibular Skeletal***										
Co displacement (mm)	AP	−2.48	−3.15	3.55	−2.36	−1	4	0.12	−0.80;2.40	0.756
	SI	−6.31	−6.6	4.4	−4.67	−3.2	3.32	1.64	−1.00;4.30	0.425
	3D	7.76	7.95	5.2	5.85	5.25	5.85	1.91	−0.89;0.10	0.244
Co-Go (mm)		4.76	4.6	2.77	4.24	3.4	3.5	0.52	−2.60;0.70	0.183
Co-Pog (mm)		5.99	6.55	4.63	5.04	4.15	6.65	0.95	−3.50;1.30	0.287
Go-Me (mm)		2.94	2.6	3.88	2.98	2.8	5.02	−0.04	−2.00;1.70	0.965
Co-Go-Gn (°)		1.96	1.15	1.5	1.65	1.35	2.07	0.31	−0.70;0.80	0.956
***Mandibular Dental***										
Lower Incisor, L1 (mm)	AP	0.92	1.2	3.23	2.45	2.55	2.13	−1.53	0.10;3.00	**0.037**
Lower right molar, LR6 (mm)	AP	2.36	2.9	2.55	3.02	3.05	1.65	−0.66	−0.49;1.60	0.308
	SI	2.06	1.7	2.4	2.51	2.4	1.88	−0.45	−0.60;1.40	0.34
Lower left molar, LL6 (mm)	AP	2.58	2.75	3.23	2.56	2.7	2.03	0.02	−1.50;1.60	0.903
	SI	1.92	1.75	2.47	2.4	2.1	2.85	−0.48	−0.69;1.40	0.499
L1 Bucco-Lingual (°)		4.67	5.85	12.9	7	8.05	9.57	−2.33	−2.40;7.30	0.458
LR6 Mesio-Distal (°)		−0.23	−1.05	7.78	2.39	3.5	6.4	2.16	0.20;6.40	**0.039**
LL6 Mesio-Distal (°)		0.69	−0.8	7.38	3.7	3.6	5.13	−3.01	0.19;6.20	**0.039**

*Mann Whitney U test was utilized to assess the difference between the two groups; statistical significance at *p* < 0.05

**Fig. 7:**
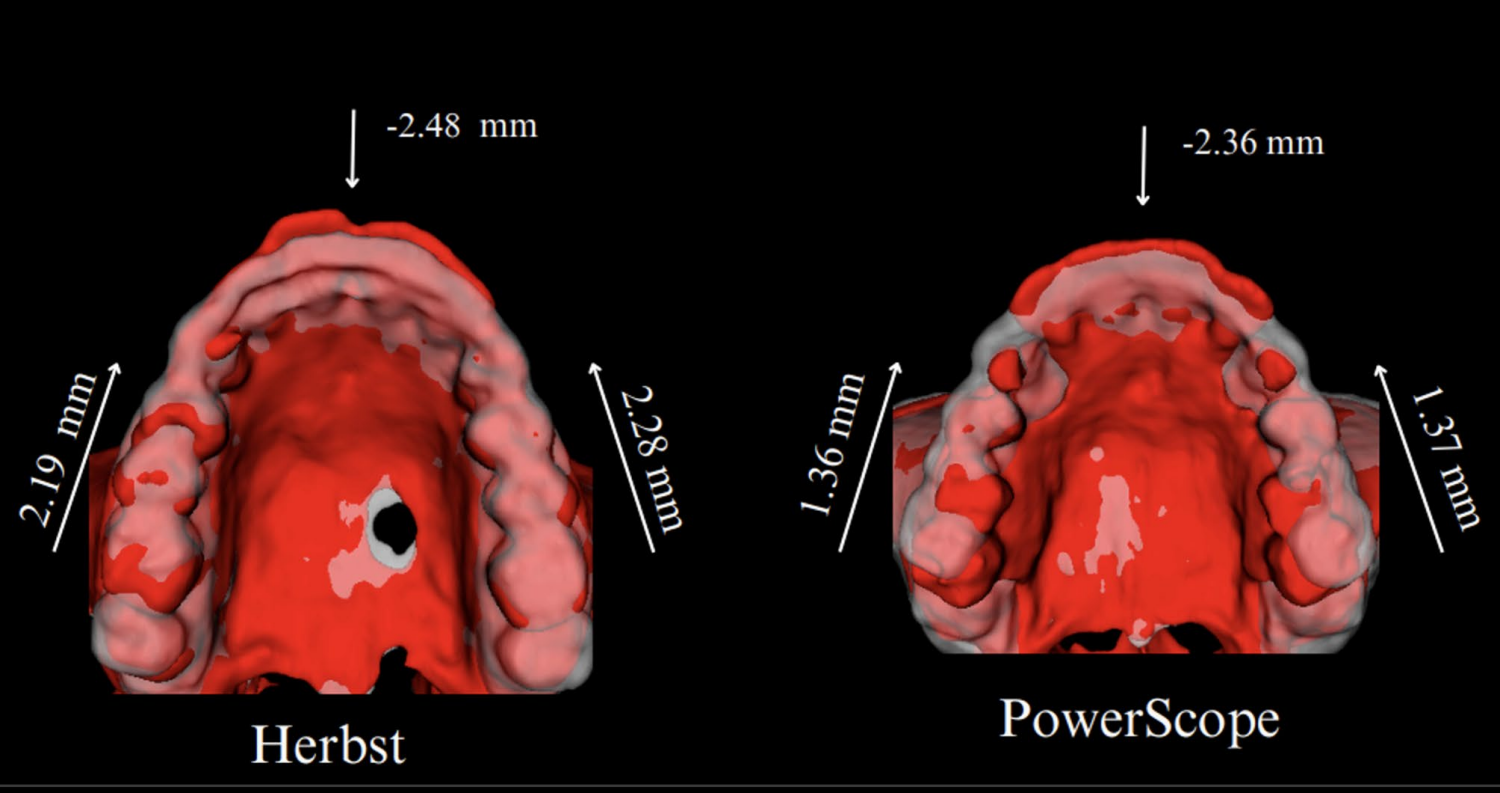
Comparison of maxillary dental changes (T1, T2) relative to the maxillary regional superimposition for the Herbst and PowerScope groups in cases representative of the sample mean values for upper molars (right and left) and upper incisors AP displacements

Maxillary changes shown in Table 6; Fig. 7 found no significant differences between groups. Both groups presented significant distal movement of the upper incisors (~ 2 mm in both groups) and mesial movement of the upper molars (approximately 2 mm in the Herbst group and 1 mm in the PowerScope group).

**Table 6:** Comparison of maxillary dental changes (T1, T2) relative to the maxillary regional superimposition for the Herbst and PowerScope groups

Variables		Herbst			PowerScope			Intergroup difference			
		Mean	Median	IQR	Mean	Median	IQR	Mean	95% CI	*p*-value^*^	
Upper Incisor, U1 (mm)	AP	−2.48	−3.15	3.55	−2.36	−1	4	0.12	−0.80;2.40	0.756	
Upper right molar, UR6 (mm)	AP	2.19	2.05	2.55	1.36	0.95	2.5	0.83	−2.20;0.20	0.108	
	SI	0.13	−0.15	2.58	0.32	0.1	1.92	−0.19	−0.80;1.20	0.665	
Upper left molar, UL6 (mm)	AP	2.28	1.8	3.65	1.37	1.1	1.98	0.91	−2.20;0.60	0.268	
	SI	0.37	0.15	1.73	0.22	0.3	1.88	0.15	−1.10;0.70	0.773	
U1 Bucco-Lingual (°)		−0.58	−0.7	7.22	4.3	1	14.2	−3.72	−2.30;10.10	0.24	
UR6 Mesio-Distal (°)		1.1	−0.95	6.7	−0.37	−0.3	4.7	0.8	−3.40;2.60	0.842	
UL6 Mesio-Distal (°)		1.02	0.45	9.25	0.49	0.4	4.33	0.53	−3.90;2.70	0.782	

Mann Whitney U test was utilized to assess the difference between the two groups; statistical significance at *p* < 0.05

**Fig. 8:**
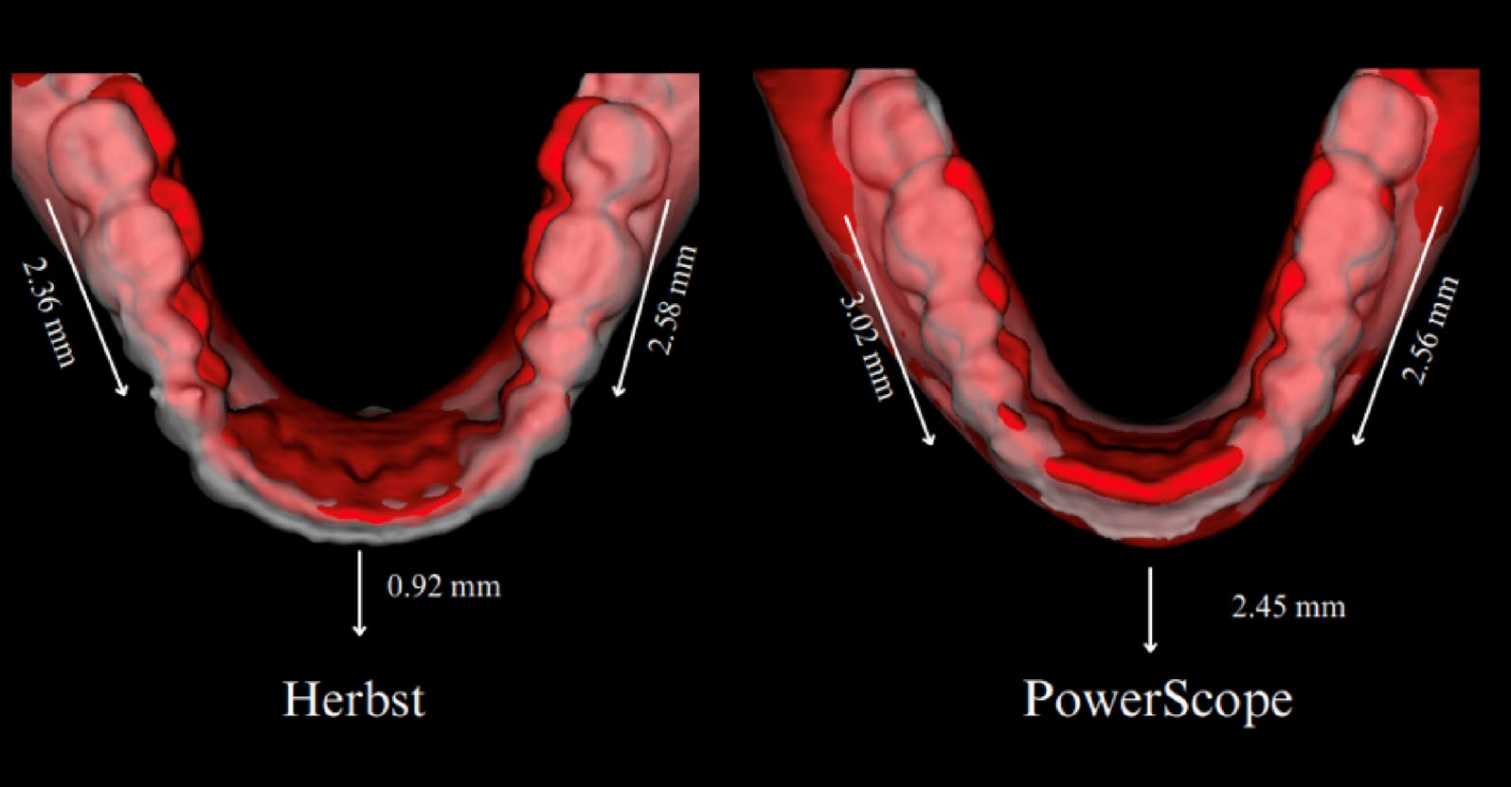
Comparison of mandibular dental changes (T1 to T2) for the Herbst and the PowerScope groups in cases representative of the sample mean values for lower molars (right and left) and lower incisors AP displacements

Figure 3 illustrates semitransparent overlays of 3D surface models superimposed at the cranial base and mandibular regions for superimposition analysis within the Herbst group. Figure 4 showcases analogous superimpositions within the PowerScope group. Figures 5, 6, 7 and 8 summarize the most clinically significant mean values observed for the maxillary, mandibular, skeletal, and dental variables.

## Discussion

### Key results and interpretation

This study tries to elucidate the mechanisms and efficacy of Class II correctors. It is the first study to compare Class II correction achieved by the PowerScope and Herbst MiniScope appliances after comprehensive orthodontic treatment, utilizing automated and open-source 3D image analyses. Previous investigations have primarily examined various designs of the Herbst appliance [7–10], while studies on the PowerScope have been limited to 2D images [6, 11]. The application of validated 3D voxel-based superimpositions on the cranial base [15], as well as regionally on the maxilla [18] and mandible [15], is now enhanced by automated AI-based approaches. This advancement enables a quantitative analysis of the skeletal and dental changes induced by each appliance.

The outcomes of our analysis distinctly underscore the differential impacts on maxillary and mandibular development, alongside alterations in the positioning of the dental arches, providing robust evidence for the selection of orthodontic appliances and strategic treatment planning. While transverse changes were not significant and were thus not reported in the present study, the 3D approach enabled more detailed visualization of craniofacial structures, eliminated structural superimpositions and magnification errors inherent in 2D analysis, and captured the topographical complexity lost when analyzing two-dimensional scans (Figs. 3, 4, 5, 6, 7 and 8).

Although both appliances effectively corrected the Class II malocclusion, the Herbst group had a significant more pronounced anterior displacement of B-point and Pog. This finding aligns with some previous studies [20, 21] but contrasts with others [10, 22–24]. Discrepancies between studies may be attributable to variations in sample characteristics, appliance design, treatment protocols, landmark definitions, and examiner inconsistencies across research centers [20, 25, 26] The Herbst group exhibited greater increases (not statistically significant) in ramus height, mandibular length, and superior-posterior condylar displacement, potentially indicative of the capacity of the appliance to allow mandibular growth to reach its full potential, particularly with a longer functional phase.

In contrast with other studies, there was no difference between the PowerScope and the Herbst appliance regarding the anteroposterior position of the maxilla as seen in the SNA angle [11, 27]. Although the Herbst appliance is designed to stimulate mandibular growth, particularly in the condylar region [7, 28], some studies suggest that its effects are more dentoalveolar than skeletal [29] and that long-term mandibular propulsion may accelerate rather than enhance growth [30]. This study lacks an untreated control group to compare the changes due to growth, but including such a group would not be feasible due to ethical reasons.

Conversely, the comparable magnitude of mandibular growth between groups indicates that the enhanced anteroposterior mandibular displacement observed in the Herbst group likely results from appliance-induced modification of growth direction rather than differential growth stimulation. The significant mandibular skeletal changes observed in both groups may be attributable to the timing of treatment, which coincided with the pubertal growth spurt, as recommended by Franchi et al. [31]

An essential consideration in treating Class II malocclusion with Herbst and PowerScope appliances is the difference in treatment duration, which is directly related to their modes of action. The Herbst appliance advances the mandible, consequently shifting anteriorly the condyle within the mandibular fossa. Theoretically, the appliance should only be removed after the condyle has returned to its initial position within the fossa. While studies [32, 33] indicate that the condyle returns to its original position in the mandibular fossa within 8 months, we preferred to extend the treatment period to 12 months to prevent any relapse associated with the remodeling of the condyle and mandibular fossa. Some studies have shown that the length of mandibular advancement is a critical factor for the maturation of newly formed bone and the stability of treatment outcomes [34, 35]. Delayed appliance removal can prevent minor residual growth and facilitate proper maturation of the newly formed bone matrix [34]. Tomblyn et al. [35] suggested using the Herbst appliance for 18 months. However, this approach may significantly extend the overall treatment length for Class II correction, given that the second phase, involving fixed appliances, may require 12 to 24 months.

Like other hybrid appliances, including Forsus and Twin Force, PowerScope is designed to promote tooth movement by applying continuous elastic force 24 h a day. These appliances share a common mechanism: using open-coil springs to generate forces ranging from 150 to 260 g. The primary goal of hybrid appliances is not to reposition the mandible anteriorly. Evidence from the literature [6, 11] suggests hybrid appliances induce greater tooth movement, likely because they do not displace the condyle from the mandibular fossa. Therefore, treatment length is determined by the time required to correct the Class II molar relationship, typically within 4 to 6 months.

Regarding the observed dental changes, both appliances effectively corrected Class II malocclusion through the mesial movement of the lower molars, with the PowerScope group exhibiting small but statistically significant mesial tipping not observed in the Herbst group (Figs. 7 and 8). Similarly, both appliances caused lower incisor proclination, but the effect was more pronounced in the PowerScope group (Fig. 8). In the upper arch, both groups presented similar levels of upper incisor retraction (approximately 2 mm). In the lower arch, the PowerScope group demonstrated significantly greater anterior displacement of the lower incisor tip and flaring of the lower incisors. The lower first molars also tipped mesially to a significantly greater extent in the PowerScope group, and both groups exhibited mesial and extrusive displacement of the lower first molars.

It is also relevant to ask whether the mechanics employed during the treatment may help explain the differences in outcomes. Class II correction was initially achieved in Herbst-treated patients, resulting in a Class III molar relationship and upper arch spacing due to molar distalization induced by the Herbst appliance. Following Herbst treatment, a fixed appliance was used to align the teeth. Upper anterior retraction led to the incisor’s distalization and anchorage loss (mesialization) of upper molars. In PowerScope-treated patients, leveling was performed first, followed by Class II correction. Because the appliance was attached to all upper teeth, the creation of larger spaces was unnecessary, as observed in Herbst-treated patients, leading to reduced anterior retraction. Nevertheless, these factors were probably not the primary cause of skeletal differences. Still, they may have contributed to dental changes, including the larger protrusion of lower incisors in the PowerScope group, as the appliance is directly anchored to the lower arch. As a result, hybrid appliances induce larger protrusion of lower incisors compared to the Herbst appliance.

The biomechanics of the Herbst and PowerScope appliances should be considered by orthodontists when selecting the most appropriate Class II corrector. It is also essential to consider the differences in fabrication (whether a laboratory phase is required) and the learning curve for their installation and management of clinical challenges that may arise during treatment. Patient adaptation to correctors is also relevant, but this subject will be dealt with in a future publication.

Finally, the decision to include superimpositions of two cases for each appliance was made to illustrate individual treatment variations clearly, provide clinicians with more precise information, and prevent any misinterpretation of the effects of the appliances (Figs. 3 and 4). The authors agree that utilizing an average perfilogram for both groups would be the most appropriate. However, generating such an image from CBCT scans is not currently feasible. Thus, we used images from cases that most accurately represent our findings.

Overall, the effective correction of Class II malocclusion observed in both groups can be attributed to favorable growth patterns and well-positioned incisors at baseline. However, treatment-induced dental changes in the symphysis could affect long-term periodontal health, highlighting the importance of individualized treatment planning. Our findings indicate that the Herbst MiniScope appliance may be particularly effective in cases requiring pronounced mandibular advancement. In contrast, the PowerScope appliance may be advantageous when dental compensation is acceptable or preferred, underscoring the importance of appliance selection based on individual patient needs. Future research should build upon the foundation established by this study, investigating the long-term stability of corrections achieved with PowerScope and Herbst MiniScope appliances and their potential differential effects on periodontal health. Additionally, further investigation into the biomechanical principles underlying the observed skeletal and dental changes could refine our understanding of appliance design and function, potentially developing more efficient and patient-friendly treatment options for Class II malocclusion.

### Limitations

The present study has some limitations that should be acknowledged and considered when interpreting the results.

Although all patients included in the study were in the same stage of skeletal growth, as assessed by the cervical vertebral maturation method, an uneven distribution of sexes was observed between the analyzed groups — with a predominance of females in the PowerScope group. Even though the literature suggests that the response to treatment with functional orthopedic appliances, such as the Herbst and PowerScope, does not vary significantly between sexes, this asymmetry in group composition may be considered a methodological limitation, as uncontrolled individual biological differences could influence clinical outcomes.

Another significant limitation is the non-randomized, retrospective design of the study. Patients were not randomly allocated to treatment groups. However, it is crucial to note that the groups were created based on the clinical examination of several patients. Approximately 300 patients were evaluated for each group, and the patients were chosen based on the criteria mentioned. The grouping did consider the device that the patient would use.

This study also lacks an untreated control group to compare the changes due to growth, but including such a 3D group would not be feasible due to ethical issues. However, it is essential to clarify that the classical studies involving untreated patients were based on lateral cephalometric radiographs, which rely on two-dimensional (2D) measurements. In contrast, the present study employed three-dimensional (3D) evaluations, including superimpositions on the cranial base, maxilla, and mandible, providing a more accurate and spatially comprehensive assessment of the skeletal and dental effects induced by the Herbst and PowerScope appliances. Due to the methodological incompatibility between 2D and 3D measurements—particularly concerning spatial displacements and volume quantification—we believe that using 2D data as a control would lead to inaccurate and potentially misleading comparisons. Nevertheless, we acknowledge the lack of a contemporary untreated control group as a limitation of our study. However, we do not believe this undermines the validity of our findings, as all comparisons were performed within a consistent and controlled 3D framework with matched skeletal maturation stages.

### Generalizability (external validity) of the study results

The results of this study can be expected to apply to child-adolescent patients with Class II malocclusion, and markers of growth spurt, particularly CVM and puberty markers for female patients. Clinician’s experience with assessing the best time for treatment, experience with the installation and maintenance of the appliances are likely important determinants of the applicability of our findings.

## Conclusions

Both appliances efficiently corrected Class II malocclusion. Herbst-treated patients exhibited more significant mandibular forward changes relative to the cranial base. PowerScope-treated patients demonstrated more significant dental changes, especially in the anterior lower arch during the pubertal growth spurt. The choice between these appliances should be based on individual patient characteristics and treatment goals.

## Data Availability

No datasets were generated or analysed during the current study.
